# Measurement of Reverse Triiodothyronine Level and the Triiodothyronine to Reverse Triiodothyronine Ratio in Dried Blood Spot Samples at Birth May Facilitate Early Detection of Monocarboxylate Transporter 8 Deficiency

**DOI:** 10.1089/thy.2020.0696

**Published:** 2021-09-07

**Authors:** Hideyuki Iwayama, Hiroki Kakita, Masumi Iwasa, Shinsuke Adachi, Kyoko Takano, Masahiro Kikuchi, Yasuko Fujisawa, Hitoshi Osaka, Yasumasa Yamada, Akihisa Okumura, Khemraj Hirani, Roy E. Weiss, Samuel Refetoff

**Affiliations:** ^1^ Department of Pediatrics, School of Medicine, Aichi Medical University, Nagakute, Japan.; ^2^ Department of Perinatal and Neonatal Medicine, School of Medicine, Aichi Medical University, Nagakute, Japan.; ^3^ Department of Pediatrics, Fukuchiyama City Hospital, Kyoto, Japan.; ^4^ Center for Medical Genetics, Shinshu University Hospital, Nagano, Japan.; ^5^ Department of Pediatrics, Hitachi General Hospital, Hitachi, Japan.; ^6^ Department of Pediatrics, School of Medicine, Hamamatsu University, Hamamatsu, Japan.; ^7^ Department of Pediatrics, Jichi Medical School, Shimotsuke, Japan.; ^8^ Department of Medicine, Miller School of Medicine, University of Miami, Miami, Florida, USA.; ^9^ Departments of Medicine, Pediatrics and Committee on Genetics, The University of Chicago, Chicago, Illinois, USA.

**Keywords:** dried blood spot, LC-MS/MS, MCT8 deficiency, newborn screening, reverse T3

## Abstract

***Background:*** Monocarboxylate transporter 8 (MCT8) deficiency is an X-chromosome-linked neurodevelopmental disorder resulting from impaired thyroid hormone transport across the cell membrane. The diagnosis of MCT8 deficiency is typically delayed owing to the late appearance of signs and symptoms as well as the inability of standard biomarkers of neonatal screening to provide early detection. In this study, we report, for the first time, the ability to detect MCT8 deficiency at birth using dried blood spot (DBS) samples.

***Methods:*** We retrospectively measured triiodothyronine (T3), thyroxine (T4), and reverse T3 (rT3) levels in DBS samples obtained at 4–5 days of life from 6 infants with genetically confirmed MCT8 deficiency and from 110 controls. The latter consisted of 58 healthy term neonates obtained at the same time, 16 were stored for more than 1 year before measurement to match samples from the MCT8-deficient infants. Ten DBS samples were collected at day 1 of life and 42 samples were from prematurely born neonates. Measurements were carried out in extract from eight millimeters diameter DBS using liquid chromatography-tandem mass spectrometry.

***Results:*** Contrary to characteristic iodothyronine abnormalities of MCT8 deficiency during later life, T3 and T4 values were not discriminatory from those of other study groups. In contrast, rT3 was significantly lower. The T3/rT3 ratio was higher in the DBS samples from the MCT8-deficient infants compared with all other groups with no overlap (*p* < 0.0001).

***Conclusions:*** rT3 and T3/rT3 ratio in DBS samples obtained from neonates can serve as biomarkers to detect MCT8 deficiency at birth.

## Introduction

Monocarboxylate transporter 8 (MCT8) deficiency (OMIM 300095) is an inherited disorder that causes severe psychoneuromotor defects with characteristic abnormalities in serum thyroid hormone (TH) function tests (1). It is caused by mutations in the *MCT8* (*SLC16A2*) gene, on Xq13.2, which impair the transport of TH into the brain, producing the psychoneuromotor abnormalities. However, excess TH available to peripheral tissues, through alternative TH transporters, produces hypermetabolism. The former abnormalities consist of early poor head control and truncal hypotonia, followed by inability to stand or walk, absent speech, abnormal involuntary movements, and spastic quadriplegia. These signs are not evident until later in life, and given the X-chromosome linked inheritance, carrier mothers are asymptomatic.

In children aged four months or more, TH abnormalities consist of high serum triiodothyronine (T3), low normal thyroxine (T4), and low reverse T3 (rT3) with normal or slightly elevated thyrotropin (TSH). Although this combination of thyroid hormone abnormalities is characteristic of MCT8 deficiency, current neonatal detection programs will not detect affected newborns. Current screening programs only measure TSH and/or T4 and will miss the high T3 and low rT3. As carrier females are asymptomatic and do not have the characteristic TH test abnormalities, prenatal diagnosis is practicable only in those women who have previously given birth to an affected child. Newborns of women not known to be carriers of an *MCT8* gene mutation may harbor embryos with a *de novo* mutation, which will not be identified until neurodevelopmental abnormalities are identified later in infancy or even childhood. This is unfortunate as a recent trial with the thyroid analogue Triac suggested that early treatment is more likely to be effective in improving or preventing the evolution of the neuromotor deficit (2).

While the thyroid tests abnormalities in MCT8 deficiency are virtually pathognomonic in both humans (1) and in mice (3,4), this may not be the case at birth. Indeed, at birth, Mct8-deficient mice have higher rather than lower serum T4 concentration and undetectably low T3 (5). Thus, we performed a retrospective pilot study to determine the nature of iodothyronine abnormalities in the first five days of life and to assess whether they could be used in the early detection of MCT8 deficiency.

In this retrospective study, we measured T3, T4, and rT3 levels in dried blood spot (DBS) samples obtained from newborns later confirmed genetically to have MCT8 deficiency. Results were compared with those from DBS samples obtained from normal controls at 1–6 days of life, in prematurely born, and in samples stored up to 2 years. To the best of our knowledge, this is the first study that measured T3 and rT3 levels in DBS specimens obtained in the first week of life. Our findings provide promising biomarkers for the early detection of MCT8 deficiency and a possible means to determine the prevalence of this devastating condition.

## Materials and Methods

### Subjects

In Japan, DBS samples are obtained from all neonates at 4–5 days of life as part of mass screening program for inborn errors of metabolism. All DBS samples in this study had TSH values within the reference range and were not recalled by the respective screening programs. The deidentified residual DBS samples, stored at room temperature, were used for our studies. There were six samples from infants with genetically confirmed MCT8 deficiency [c.733C>T (p.R245X); c.1556C>A (S519L); c.883G>A (p.G295S); c.985_986insG (p.Asp329Glyfs*1) c.661G>A (p.G221R); and c.1188dup (p.Ile397Hisfs*57); amino acids numbered according to the long form of the MCT8 protein]. As controls, 110 DBS samples were obtained consisting of healthy neonates, 16 of which were measured after storage for more than a year. In addition, 10 DBS samples were obtained from healthy infants born at term but collected at the end of the 1st day of life, as this is sometimes the practice in the United States for neonates and mothers discharged 1 day postpartum. Of the total of 106 DBS samples, 88 were collected in Japan and 18 in the United States. The same filter paper was used. Details on demographics including gestational age, sex, birth weight, and duration of sample storage before analysis are provided in Supplementary Table S1. The study was approved by the ethical committees and institutional review boards of the three involved institutions (Aichi Medical University, 2015-H359; Universities of Miami and Chicago, 20200996). A written informed consent was obtained from the parents of all study subjects. All study evaluations and procedures were performed in accordance with the Declaration of Helsinki.

### Methods

T3, T4, and rT3 were measured by liquid chromatography-tandem mass spectrometry (LC-MS/MS) as described previously (6). It was based on the LC-MS/MS method used in mouse serum and tissues, and results were in agreement with those obtained by radioimmunoassay (5). The LC-MS/MS method was modified for use in DBS samples (for details see Supplemental Data).

### Statistical analysis

T3, T4, and rT3 levels are expressed as mean ± standard deviation (SD). Statistical analysis using analysis of variance with Dunnett's test for comparison of differences between all five groups was performed at Aichi Medical University and results were confirmed at the University of Chicago.

## Results

The demographics of the two groups are given in Supplementary Table S1. These were not significantly different with respect to gestational age or birth weight except for the group of prematurely born. The same table provides information regarding age at blood sampling and the time elapsed from sampling to analysis. As storage is expected to produce iodothyronine degradation (7) by deiodination, a group of DBS samples obtained from normal infants but stored for 1.1–2.2 years were included to match the stored DBS samples to the MCT8-deficient group.

**FIG. 1.**
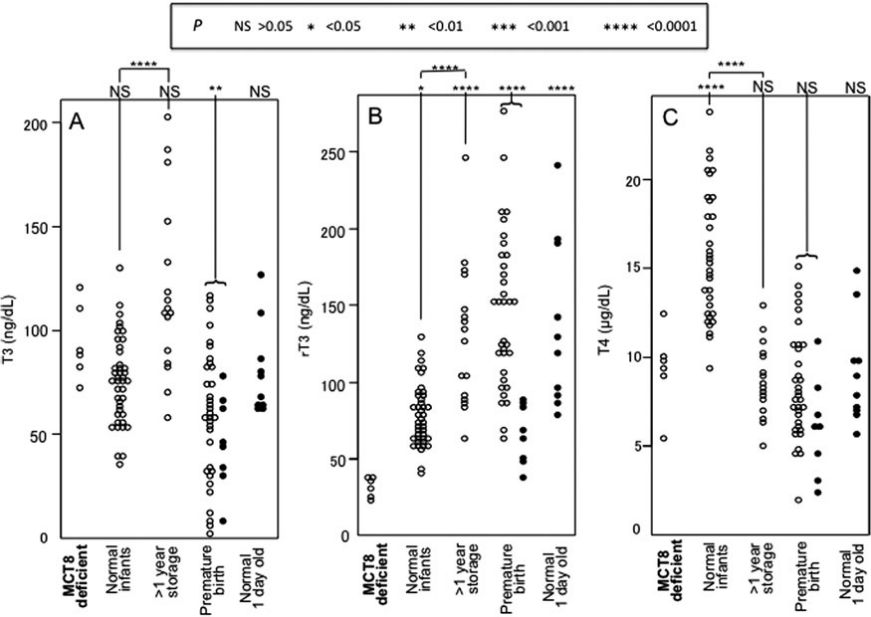
Concentrations of T3 **(A)**, rT3 **(B)**, and T4 **(C)** in individual DBS samples in groups identified on the bottom of each panel. *p*-Values obtained by comparisons using ANOVA between samples from MCT8-deficient and each other group are given on top of each panel. The *p*-values of differences between the samples from normal neonates one of which was stored for more than 1 year (1.1–2.3 years) are also shown. Open circles identify samples collected in Japan and closed circles identify samples collected in the United States. ANOVA, analysis of variance; DBS, dried blood spot; MCT8, monocarboxylate transporter 8; rT3, reverse T3; SD, standard deviation; T3, triiodothyronine; T4, thyroxine.

**FIG. 2.**
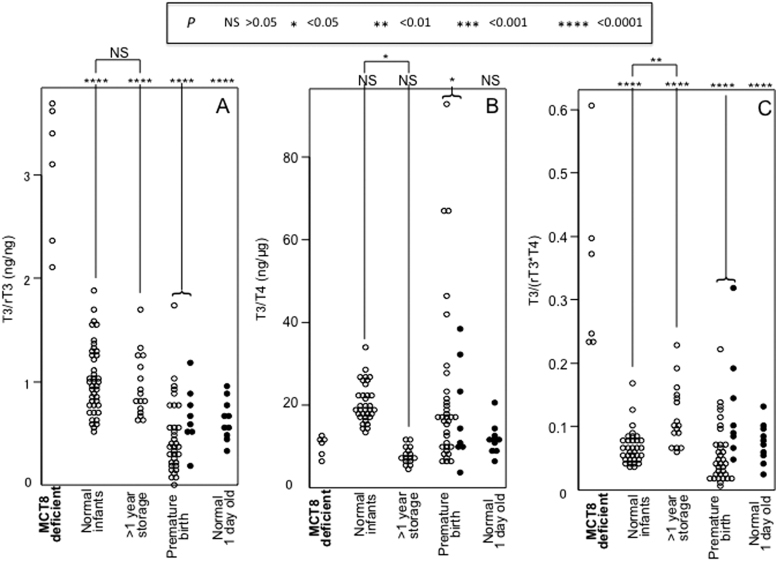
Calculated T3/rT3 **(A)**, T3/T4 **(B)**, and T3/(rT3 · T4) **(C)** ratios in individual DBS samples in groups identified on the bottom of each panel. *p*-Values obtained by comparisons using ANOVA between samples from MCT8-deficient and each other group are given on top of each panel. The *p*-values of differences between the samples from normal neonates one of which was stored for more than 1 year (1.1–2.3 years) are also shown. Open circles identify samples collected in Japan and closed circles identify samples collected in the United States.

Results of the study are presented in Figures 1 and 2, and mean values ± SD are provided in Table 1. The concentrations of T3, rT3, and T4 are shown in Figure 1A–C. The T3 concentrations of the MCT8-deficient infants were not significantly different than those of the other groups, except for the group of prematurely born, whose T3 was lower (57 ± 30 vs. 94 ± 18; *p* < 0.01; Fig. 1A). The concentrations of rT3 in the MCT8-deficient newborns were significantly lower than that of all other groups (Fig. 1B; mean values ± SD in Table 1). The concentration of T4 was also not significantly different than the other group, except for those of the normal infants (Fig. 1C; Table 1). Of note, taking into account the effect of storage, the T4 concentrations in DBS samples of MCT8-deficient infants were not significantly different compared with the normal infants.

**Table 1.** Number of Infants Analyzed, Thyroid Hormone Concentrations, and Ratios

		Mean ± SD					
		T3	rT3	T4	T3/rT3	T3/T4	
Group	N	ng/dL	ng/dL	μg/dL	ng/ng	ng/μg	T3/(rT3 × T4)
MCT8 deficient	6	94 ± 18	32 ± 7	9.3 ± 2.2	3.04 ± 0.67	10.1 ± 2.3	0.349 ± 0.146
Normal infants	42	76 ± 21	78 ± 20	15.7 ± 3.6	1.02 ± 0.34	20.5 ± 4.7	0.067 ± 0.027
Normal infants (DBS stored >1 year)	16	119 ± 42	130 ± 47	8.7 ± 2.1	0.96 ± 0.31	7.8 ± 2.2	0.117 ± 0.049
Prematurely born	34 + [8]	57 ± 30	131 ± 56	7.9 ± 3.1	0.50 ± 0.35	21.1 ± 18.4	0.071 ± 0.063
Normal infants (DBS collected at day 1)	[10]	80 ± 22	137 ± 55	9.2 ± 3.0	0.64 ± 0.20	11.8 ± 3.8	0.075 ± 0.031

Numbers in square brackets are samples from the United States.

DBS, dried blood spot; MCT8, monocarboxylate transporter 8; N, number of DBS; rT3, reverse T3; SD, standard deviation; T3, triiodothyronine; T4, thyroxine.

We calculated the ratios of T3/rT3, T3/T4, and T3/(rT3 × T4). The T3/rT3 and T3/(rT3 × T4) ratios between the MCT8-deficient and all other groups were significantly different (Figs. 1A and 2C; *p* < 0.0001). The values of the MCT8-deficient infants did not overlap with those of the other groups. Of note, the T3/T4 ratios between the MCT8-deficient group and the corresponding matched normal controls of newborns, whose DBS samples were stored for more one year, completely overlapped (Fig. 2B). Ratios of T3/(rT3 + T4) (not shown) did not give better discriminatory results.

## Discussion

Currently, most neonatal screening programs are based on measurement of TSH only in DBS samples and, therefore, have not detected newborns with MCT8 deficiency. Programs that also measure T4, total or free, have picked up the low concentration in some newborns with MCT8 deficiency. This led in some instances to treatment with physiological doses of levothyroxine, with no beneficial effects (8,9). In fact 6 and 4 of 8 DBS samples from newborns with MCT8 deficiency had total T4 values that were 1 and 2 SDs, respectively, below the mean value (10). In another report of 8 MCT8-deficient newborn, the mean total T4 was 5.1 ± 1.6 μg/dL with a range of 3.1–8.4, compared with a reference range of 6–15 μg/dL (11).

Serum TH tests abnormalities in older infants, children, and adults with MCT8 deficiency are typical if not fully pathognomonic. However, it could not be construed that this will also be the case in the first few days of life, given their immature livers, and the contribution of other tissues to the metabolism of TH affecting serum TH concentrations. Based on studies in adult mice with Mct8 deficiency, serum T4 is low due to the combination of reduced secretion from the thyroid gland, increased consumptive degradation by deiodinase 1, and increased excretion in urine (12,13). Serum T3 is increased owing to the increased conversion of T4 to T3 by deiodinases 1, its decreased degradation by low deiodinase 3 activity, and, to some extent, reduced transport into tissues (3,4). The high deiodinase 1 and low deiodinase 3 activity contribute to the markedly reduced rT3 (14). While Mct8-deficient mice manifest the same abnormalities as humans during adulthood, this is not the case in the early postnatal life. Postpartum low rT3 is the first to manifest, followed by low T4, high TSH, and only then high T3 (3,5). However, in humans with MCT8 deficiency, the TH abnormalities during the first few days of life remained unknown until this study. Thus, the primary aim of this study was to determine the nature of iodothyronine abnormalities in the 1st week of life. Should MCT8-deficient newborns present characteristic TH abnormalities similar to those found in later life, they could be applied to possibly detect MCT8 deficiency. A future screening program using such tests would enable the determination of the prevalence of this defect.

To the best of our knowledge, this is the first study that has measured T3 and rT3 levels in DBS samples obtained at birth. MCT8-deficient newborns showed significantly decreased rT3 and T4 compared to normal newborns. Measurement of T3 in the MCT8 deficient newborns was not significant from normal newborns as the elevated T3 is seen only in later life (Fig. 1). This is not surprising as T3 is maintained low in the fetus. However, the rT3, normally elevated at birth, was significantly reduced in MCT8-deficient newborns. More importantly the ratios of T3/rT3 and T3/(rT3 × T4) were lower and with no overlap with all other groups (Fig. 2). The values of the latter two in control, compared with MCT8-deficient newborns, did not overlap (Fig. 1). This suggests that these biomarkers could have 100% sensitivity and specificity for the detection of newborns with MCT8 deficiency.

Newborns T4 and FT4 concentrations can be low in prematurity, critical illness, thyroglobulin deficiency, and in infants born to thyrotoxic mothers as well as in congenital central hypothyroidism (15,16). In contrast, in embryonic fluids and at birth, T3 is low and rT3 is high (17,18). In congenital central hypothyroidism, both T3 and rT3 are low (18). Thus, measurement of all three iodothyronines may be necessary to identify newborns with MC8 deficiency. LC-MS/MS provides these results at the same time without additional effort or cost.

The key prerequisites for an effective neonatal screening program include adequate knowledge of the natural history, availability of methods for early diagnosis, and effective treatment to improve prognosis (19). The natural history of MCT8 deficiency is well known (10). This study provides a basis for the development of neonatal screening test for MCT8 deficiency. The use of LC-MS/MS in routine clinical diagnosis is gaining popularity and has already become the preferred method in the measurements of serum testosterone and thyroglobulin. With reduction in cost of LC-MS/MS measurement and the increase in survival and cost for the care of individuals with MCT8 deficiency, the application of LC-MS/MS in neonatal screening is possible. Although current treatments of MCT8 deficiency are at best palliative, more effective early treatment using TH analogues (2) and gene-directed treatments are under development (11,20).

In conclusion, measurement of rT3 and the T3/rT3 ratio in DBS samples at birth are useful for early detection of MCT8 deficiency and will help determine the prevalence of this condition.

## Supplementary Material

Supplemental data

Supplemental data
