# Supplementary material for: Measurement of Reverse Triiodothyronine Level and the Triiodothyronine to Reverse Triiodothyronine Ratio in Dried Blood Spot Samples at Birth May Facilitate Early Detection of Monocarboxylate Transporter 8 Deficiency
Source: Thyroid. 2021 Sep 7;31(9):1316–21. doi: 10.1089/thy.2020.0696 (PMC8558056; doi:10.1089/thy.2020.0696)
Supplement: Supplemental data [file Supp_TableS1.docx]

Supplemental Table 1. Demographics of and DBS collection and measurements

.

Sex Gestational Birth Age at DBS Time of measure-

Group N age weight collection ment after collection

M/F weeks g days (range)

.

MCT8 deficient 6 6/0 40.1 ± 1.9 3173 ± 436 4 - 5 3.4 ± 1.5 yr

(1.4 – 5.3)

Normal infants 42 19/23 39.6 ± 1.3 3063 ± 346 4 - 5 5.5 ± 1.3 mo

(2.8 – 7.7)

Normal infants 16 5/11 40.0 ± 1.1 3235 ± 385 4 - 5 1.4 ± 0.4 yr

(DBS stored >1yr) (1.1 – 2.3)

Prematurely born 34 + [8] 22/20 32.6 ± 3.6 1841 ± 611 3 - 6 2.1 ± 1.3 mo

(0.4 – 5.2)

Normal infants [10] 4/6 39.1 ± 1.1 3290 ± 644 1 1.3 ± 0.3 mo

DBD collected at day 1 (0.7 – 1.7)

.

Numbers in square brackets are samples from USA; Values are mean ± SD and ranges in brackets. yr, years; mo, months
