# Supplementary material for: Measurement of Reverse Triiodothyronine Level and the Triiodothyronine to Reverse Triiodothyronine Ratio in Dried Blood Spot Samples at Birth May Facilitate Early Detection of Monocarboxylate Transporter 8 Deficiency
Source: Thyroid. 2021 Sep 7;31(9):1316–21. doi: 10.1089/thy.2020.0696 (PMC8558056; doi:10.1089/thy.2020.0696)
Supplement: Supplemental data [file Supp_Data.docx]

**SUPPLEMTAL DATA**

***Reagents and standards***

T3, T4, rT3, and their ^13^C-labeled internal standards (^13^C_6_-T3, ^13^C_6_-T4, and ^13^C_6_-rT3, respectively) were purchased from IsoScience (King of Prussia, PA, USA). The Kinetex C18 2.6-µm column (00D-4462-AN) and SecurityGuard^TM^ ULTRA cartridge (AJ0-8782) were obtained from Phenomenex (Torrance, CA, USA). Centrifuged filter with hydrophilic polyvinylidene fluoride (PVDF) membrane (Ultrafree-MC HV, 0.45 µm) was purchased from Millipore (Billerica, MA, USA).

Calibration standard solutions were prepared by spiking the delipidized human serum with ultra-low hormones, steroids, and other analytes (MSG 3000, Golden West Biologics. Inc., Temecula, CA, USA) with various amounts of stock solutions of T3, T4 and rT3 (IsoScience).

***Instruments***

The liquid chromatography-tandem mass spectrometry (LC-MS/MS) technique was developed on a LTQ Velos Pressure Linear Ion Trap LC-MS/MS system coupled to an Accela ultra performance liquid chromatography system (ThermoFisher. Waltham, MA, US) in the positive ion multiple-reaction monitoring mode. The initial conditions for the LC gradient were 50% A [0.1% formic acid in water (v/v)] and 50% B (MeOH). The composition was changed gradually over 3 minutes till a composition of 20% A and 80% B was obtained. Then, 100% B was continued for 1 min, followed by the composition of 50% A and 50% B for 1 min.

The precursor-product ion pairs of T3 (*m/z* 652→605), ^13^C_6_-T3 (*m/z* 658→611), rT3 (m/z 652→507), ^13^C_6_-rT3 (m/z 658→513), T4 (*m/z* 778→731), and ^13^C_6_-T4 (*m/z* 784→737) were selected. The spray voltage was set at 4.0 kV, and the capillary temperature was set at 450°C. The sheath gas flow rate was 10 (arbitrary units). Quantification was performed by selected reaction monitoring using peak areas.

***Sample preparation***

Specimens of DBS (diameter: 8 mm) were used to measure T3 and rT3 by LC-MSMS using the modified method reported elsewhere (1). In brief, an 8-mm circle was punched out from the DBS and was added into 200 µL of 2% NH_4_OH in EtOH (v/v). After incubation for 30 min at room temperature, the mixtures were centrifuged at 20,000 *g* for 5 min. The filter papers were washed by 50 µL of 2% NH_4_OH in EtOH (v/v). The supernatants were combined, and ^13^C_6_-labeled internal standards were added to the samples at the final concentration of 2.4 ng/mL for T3 and 1.0 ng/mL for rT3. The solution was evaporated by freeze-drying overnight. Subsequently, the residue was reconstituted using 400 µL of 2% NH_4_OH in EtOH (v/v). The pellet was washed by 100 µL of 2% NH_4_OH in EtOH (v/v). Subsequently, the combined supernatants were evaporated by freeze-drying overnight.

The residue was dissolved in 100 µL of MeOH:H_2_O:HCOOH (50:50:0.05), which was used for measuring T3 and rT3. For measuring T4, the samples used for measuring T3 and rT3 were diluted 50 times in MeOH:H2O:HCOOH (50:50:0.05). After filtration using a centrifugal filter, each 30-µL aliquot of the resulting sample was subjected to LC-MS/MS analysis for measuring T3 and rT3.

According to the preliminary experiment, a 3-mm circle did not have enough power to evaluate T3 and rT3 in DBS. Therefore, an 8-mm circle punched out from DBS was used in this study. An 8-mm circle from DBS was calculated to have 12.5 µL of serum (1).

**1.** Nakano M, Uemura O, Honda M, Ito T, Nakajima Y, Saitoh S 2017 Development of tandem mass spectrometry-based creatinine measurement using dried blood spot for newborn mass screening. Pediatr Res **82**:237-243.
